# Impediments for the Uptake of the Botswana Government's Male Circumcision Initiative for HIV Prevention

**DOI:** 10.1155/2013/387508

**Published:** 2013-10-08

**Authors:** Motshedisi Sabone, Mabel Magowe, Lesego Busang, Jonathan Moalosi, Benjamin Binagwa, Janet Mwambona

**Affiliations:** ^1^University of Botswana, Gaborone 70318, Botswana; ^2^ACHAP, Gaborone, Botswana; ^3^Ministry of Health, Gaborone, Botswana

## Abstract

Botswana remains one of the countries with high prevalence of HIV infection with a population prevalence rate of 17.6 in 2008. In 2009, the Ministry of Health launched male circumcision as an additional strategy to the already existing HIV preventive efforts. The purpose of this paper is to share what the participants of a survey to evaluate a short-term male circumcision communication strategy in seven health districts of Botswana reported as impediments for the program's uptake. Qualitative data were obtained from 32 key informants and 36 focus group discussions in 2011. Content analysis method was used to analyze data and to derive themes and subthemes. Although male circumcision was generally acceptable to communities in Botswana, the uptake of the program was slow, and participants attributed that to a number of challenges or impediments that were frustrating the initiative. The impediments were organized into sociocultural factors, knowledge/informational factors, and infrastructural and system factors.

## 1. Introduction

Like many countries in sub-Saharan Africa, Botswana continues to register high HIV prevalence rates. The country's AIDS Impact Survey III (BAIS III) of 2008 reported that the population HIV prevalence rate was 17.6 with the rates being higher for women (20.4) than for men (14.2). The rural and urban population prevalence rates were 17.1 and 17.9, respectively. The population HIV incidence rate was 2.9, while those for men and women were 2.3 and 3.5, respectively [1]. Although the country has made great strides in areas such as antiretroviral therapy, prevention of mother-to-child transmission of HIV, and orphan care, prevention of HIV infection in general still needs strengthening [2].

In 2009, the Ministry of Health launched male circumcision as an add-on strategy to already existing HIV preventive efforts such as counselling and testing, delay of sexual debut, condom use, avoidance of multiple sexual partners, and prevention of mother-to-child transmission (PMTCT). Male circumcision was targeted at HIV-negative males aged 0–49 years with the aim of reaching 80% coverage by 2016. However, it does not look like the country will have gone any mile in 2016 as in 2010, only about 11% of the targeted number had been circumcised [3]. 

The delay in reaching the targets has been partly due to the challenges that frustrate the initiative [4]. One of the issues in mobilizing communities for HIV prevention has been the sensitivity of sex, an area that is an extremely private domain. It has therefore been critical that the government remains alert to communities' response or reaction to preventive measures being promoted. During the latter part of 2009, the government launched a short-term male circumcision communication strategy, a six months multimedia campaign that disseminated information through billboards, branding of public transport buses, television, radio, and public address at strategic places. In 2011, the short-term male circumcision communication strategy was evaluated through a survey that had both the qualitative and the quantitative components. This paper is focused on the findings of the qualitative component of the evaluation and limits itself to what the respondents reported as constraints that were hindering men to turn up for circumcision, referred to here as challenges of male circumcision and its communication strategy. Other findings of the survey are reported elsewhere. Whereas the authors acknowledge the variability of the approaches to male circumcision and the age at which it is performed in different parts of the world [5], male circumcision as discussed in this paper is a surgical procedure that involves the excision of the double layered skin that normally covers the glans penis [6]. 

## 2. Purpose of the Study and Methods

### 2.1. Purpose

The purpose of the evaluation was to determine the extent to which the short-term male circumcision communication strategy was effective in informing communities about and creating a demand for male circumcision. Further, the findings of the evaluation were expected to provide lessons that would inform a long-term male circumcision communication strategy. 

### 2.2. Methods

The survey was cross-sectional and qualitative and used purposeful sampling in the selection of participants. The study was done in seven of the 29 health districts of Botswana in 2011. The seven districts were those which the African Comprehensive AIDS Partnership (ACHAP) was working with. ACHAP is a Bill and Melinda Gates Foundation that is supporting Botswana Government's response to HIV and AIDS. Permission to conduct the survey was granted by the Ethical Review Board of the Ministry of Health, Botswana, and participants of the survey provided written consent. 

Data were obtained from 32 key informants and 36 focused group (FG) discussions. Inclusion criteria were age of at least 18 years and willingness to participate. Key informants' interviews were guided by a semistructured interview guide covering participant's background, specific role of represented organization in the strategy, perceived achievements of the strategy, views about advocacy and community mobilization, and perceptions about human resources capacity to implement the strategy. The interview guide for focus group discussions covered a background about participants, knowledge and understanding of the male circumcision services, attitudes and perceptions about male circumcision, accessibility of male circumcision services, and accessibility of male circumcision communication materials. Participants were recruited through the help of government officers based in the respective districts. 

Key informants were mainly actual or potential program implementers such as doctors, nurses, and representatives of health-related community based and nongovernmental organizations. Focus group discussants were mainly actual or potential program beneficiaries such as men, women, and the youth aged 18 years or older. In each district, there was at least one group of each of the following: community leaders, circumcised men, uncircumcised men, female partners of men eligible for circumcision (circumcised and uncircumcised), and parents of male children eligible for circumcision. Interviews and group discussions were facilitated by trained interviewers, facilitators, and note takers; they were audio-taped and later transcribed verbatim to text. Key informants were interviewed in English through the help of an interview guide. Focus group discussions were conducted in Setswana, and the transcripts were coded when they were still in the vernacular language and were thereafter translated into English. 

Content analysis method was used to analyze data and to derive themes and subthemes. Two researchers cocoded the data and continuously cross-checked with one another for consistency of assigned codes. Emerging themes and subthemes were derived, and patterns were noted. 

## 3. Results, Discussions, and Implications

### 3.1. Results

#### 3.1.1. Characteristics of the Survey Participants

There were in all 36 focus groups with an average of six persons per group and five focus group discussions per district comprising community leaders, parents/guardians of male children, female partners of men eligible for circumcision, circumcised men, and uncircumcised men. The modal age range in years for focus group discussants was midtwenties to midthirties. Key informants were government implementers and strategic partners involved in other aspects of the male circumcision program such as capacity building and service provision. The average length of service for the key informants sample was 2.8 years, with 4.8 years for Gaborone (capital city of Botswana) and a range of two to three years for the rest of the districts. Males and females constituted 53% and 47% of the key informant sample, respectively. 

#### 3.1.2. Main Study Findings and Organizing Framework

Study findings reported in this paper confine themselves to the factors that the researchers identified as challenges of the male circumcision initiative and its communication strategy that were encountered by communities in the sampled district. Whereas the quantitative aspect of the study was expected to provide the extent of the effectiveness of the communication strategy, the qualitative aspect was expected to augment the findings through addressing the question ‘‘why?”. 

The impediments to the uptake of male circumcision and its communication strategy were organized into three main categories, namely, sociocultural factors, knowledge/informational factors, and infrastructural and system factors. Sociocultural factors are those related to the way of life for the communities, as well as their values and beliefs that inform their practices; the manner in which male circumcision was communicated often clashed with communities' values. Knowledge/informational factors relate to the nature and quality of male circumcision and related information that participants had; inadequate or distorted information was often a source of anxiety and uninformed conclusions. Infrastructural and system factors cover availability of resources such as health facilities, personnel, equipment, and supplies as well as an efficient and coordinated system with open lines of communication; responses on infrastructural and system factors should sensitize program implementers to examine what they do and how they do it and to identify strengths and gaps in the services being provided.

*(1)* *Sociocultural Factors*. Although generally the respondents were welcoming male circumcision as another way of addressing HIV and AIDS, it was evident that the campaign messages were received with caution lest it could disturb the existing order. Examples of areas in which some participants were not comfortable with the campaign follow.

*Gender and Age Factors*. Gender issues were reported in Borolong, Kgalagadi South, Mahalapye, and Selibe-Phikwe and were concerned with male adolescents being uncomfortable with female community mobilizers and men's discomfort in being attended by female surgeons. Men viewed female surgeons who approached male circumcision in a matter-of-fact way as disrespectful. However, some argued that it was just a mentality that men needed to wean themselves from and be as comfortable with female circumcisers as women were with male midwives. In a similar vein, older men were not comfortable being addressed by the youth on circumcision. The uncomfortable environment often denied sharing and asking, whereby people could learn more about male circumcision, and whereby some misconceptions could be dispelled.

*Potential Tension between Medical Science and Setswana Tradition over the Claim to Male Circumcision Knowledge*. Through the government's male circumcision initiative, the modern health system was said to be making a claim on the knowledge that had for time immemorial been owned by traditional leaders and ethnic groups as part of the initiation of young men into adulthood. The concern was raised in Borolong, Gaborone, and Selibe-Phikwe by women, circumcised men, and uncircumcised men. Participants observed that the government would have better outcomes if the male circumcision strategy had taken aboard the traditional initiation systems through a collaborative process. What they could foresee in the near future was competition for clients between the traditional circumcisers and the modern health care system. Communities that have been circumcising through initiation schools will be in arms against the government because we have taken over what was initially their responsibility. (FG Circumcised Men, Selibe-Phikwe).

Participants were not saying that they were against male circumcision. They saw it as a welcome development to address the prevailing challenges. Their concerns were about long-term outcomes and sustainability of the male circumcision strategy. They noted that new developments were welcome to address new challenges. “Life has changed as for instance, there are new diseases and increased number of hospitals; there are new challenges, and we should also change,” (FG Circumcised Men, Selibe-Phikwe). It was evident that participants needed to be acknowledged—for instance, that male circumcision was not really new and that it must build upon what had long been established—traditional male circumcision associated with initiation into manhood (*bogwera*).

*(2)* *Informational/Knowledge Factors:*

*Misconceptions about Male Circumcision*. Various misconceptions surrounding male circumcision were reported in all the seven (7) districts. Sometimes the potential beneficiaries of male circumcision got scared by the stories that people told them including the severity of circumcision pain. Other common misconceptions reported were impaired sexual performance and enjoyment and the belief that male circumcision provided 100% protection against HIV. It was noted that, just like the general public, some health workers had misconceptions about male circumcision and that they therefore needed more education on the subject. Regarding men who went ahead to circumcise and later scared others away by exaggerating the circumcision pain, participants noted that those who have circumcised must be advised to be cautious so as to avoid scaring away others who have interest.

*(3)* *Infrastructural and System Factors*. The districts of Borolong, Kweneng West, and Mahalapye reported facility inadequacy, shortage of personnel, and shortage of supplies. Some men had abandoned the decision to circumcise after having the surgery cancelled because of the shortage of supplies. Some had given up after waiting in long queues or schedule lists or after being told to return at a later date. In Kgalagadi South, Mahalapye, and Ngami East, treatment centers were far to reach with clients traveling long distances. There were long queues and long waiting periods which discouraged potential circumcision clients. Public transport was erratic and cumbersome in some areas. When transport was poor, coming back after failed attempt became an even more remote option.

Participants called for male circumcision services to be brought nearer to the people and for doctors to be available to avoid back-log of clients. Same date services were said to be ideal for men who need a little push for them to seek health services. Participants called upon the government to scale up the capacitation of appropriate NGOs so they could absorb some of the circumcision client load. 

Ineffective postoperative pain control was reported to be a serious deterrent for male circumcision. Pain appeared to be the single most disturbing factor about male circumcision. However, participants expressed concern that health providers in public health facilities tended to underestimate the pain associated with circumcision. They did not believe that paracetamol that was given to control pain following circumcision was strong enough for the pain and recommended that another type of medication should be used instead. Pain could be exacerbated by irritation of the wound by drops of urine, penile erection, and pressure from bed linen. It could cause difficulty in or fear of passing urine or assuming a normal gait. Some participants believed that the fact that surgery was performed when one was alert might also be contributing to the perception of pain and its unbearability. Following is an excerpt of what participants said:

*The most disturbing side effect of circumcision is pain; and something must be done about it. I am informed that people are given paracetamol. I wish there was a pain pill specifically designed for male circumcision and nothing else; not paracetamol which can be used for the ear, the nose, or any other body part…. The mere recall of the scene of what one witnessed may also make pain unbearable. I wish the person could be put to sleep when circumcision is done. (FG Uncircumcised Men, Selibe-Phikwe)*

One health care system problem noted was that it was difficult to mobilize men because the system had no programs targeting men. In fact, some participants commended the government for instituting male circumcision as the first program of its kind because it was targeting men.

*HIV Test as a Requirement for Circumcision*. There was a general consensus among the study participants that the requirement that those who sought circumcision must first go for HIV testing was deterring many men from circumcising. Participants noted that men are by nature very private people and that they usually do not like their personal lives to be exposed to public scrutiny. Men were therefore not comfortable going for HIV testing, and many were choosing to infer their HIV status from that of their female partners. The following is an excerpt of what they said:

*If the requirements of HIV testing and the … could be lifted, we would see men turning up for male circumcision in large numbers. We would have good response of men if they are required nothing else but just circumcision because even counseling delays their response. (FG Community Leaders, Selibe-Phikwe)*

#### 3.1.3. Cues to Action in Male Circumcision

Although participants reported barriers to circumcision such as pain and misconceptions, those who had circumcised shared experiences of what motivated them to reach a decision. For one man, it started with the demands of the (prevention of mother-to-child-transmission) PMTCT; PMTC demands that parents of the unborn baby test. When the couple had the first baby, the husband had ignored the wife's plea that he tested. With the second pregnancy, the wife had started asking the husband to test again. Initially, the man had not cooperated, not only because he had felt like being directed but also because he feared the positive results. This time around, there was also a talk about male circumcision. He still feared testing, but at least then he could do it as a personal choice and initiative. He was bold enough to register for male circumcision, and he went through the test, which came up negative. He then shared his HIV test results with his wife. 

Another man saw a pamphlet inviting men to go for circumcision. He developed interest. He then listened to a radio program in which an expert was being interviewed. It did not sound that scary, and his interest intensified. His mother also talked to him about circumcision, and it still sounded interesting. His younger brother had had circumcision when he was young. After some time, the man asked himself, “If my younger brother was able to do it and survive it, should I really be scared to death myself?” (FG Circumcised Men, Selibe-Phikwe). Then he decided to be bold and go for it.

One other man was encouraged by a friend who had circumcised. He then met a doctor who also motivated him. Then he sought out billboards and pamphlets for more information before he made up his mind to do it. Health professionals with the right attitude, the right information, and the right approach to carry through someone who is not only lacking information but who also has some misinformation and fears about circumcision were reported to be ideal for male circumcision information dissemination. A step-by-step explanation and individual education on the process covering what male circumcision is all about, pre- and posttreatment information, wound care, and the healing process were reported to have a high probability of increasing the demand for male circumcision.

### 3.2. Discussions

Although participants generally viewed the government's male circumcision initiative as a welcome development, they were concerned that the initiative was not attracting as many men as it had been anticipated. Infrastructural and system challenges such as long distances to treatment centers, long waiting periods, and shortage of staff and equipment could become increasingly felt as the demand for male circumcision increases and need to be given due consideration; lest they act as deterrent for clients' use of the services as it was already evident from the participants' reports. 

The challenges of male circumcision reported in this study corroborate with the findings of other male circumcision studies in other African countries such as Kenya, Malawi, and Tanzania, the majority of which have been qualitative. Only one of the studies reviewed used the quantitative approach, and like in the rest of the studies, pain and the cost of treatment were found to be important deterrents for male circumcision [7]. In Malawi, reported barriers to circumcision have included the distance to treatment sites with associated expenses, fear of wound infection, the cost of the services including time away from productive work, and pain at the time of surgery and during the healing period; especially associated with erection [8]. Pain, fear of infection, and difficulty in accessing health facilities have deterred Kenyan men from circumcising; and access has especially been a challenge in rural areas where communities have demanded that services should be brought closer to them [9]. 

Another barrier to circumcision has been misconceptions surrounding sexual pleasure and performance [9]. Similar to what was found in this study, other studies have shown issues of sexual performance and sexual pleasure as frequently reported misconceptions surrounding male circumcision. Views about sexual pleasure tend to be mixed, with some reporting that circumcision increases sexual pleasure, some claiming it improves sexual performance, while others reporting that circumcision is irrelevant to sexual pleasure and performance [8]. In one study, participants' views about the effect of male circumcision on sexual pleasure were influenced by whether one was circumcised or not, with both the circumcised and the uncircumcised being content with their situation [7]. Although gender issues did not seem to be a disturbing challenge in prior studies, a sentiment expressed by health workers in Kenya that male nurses could be trained to perform circumcision [9] may suggest the preference for male over female health providers. 

Whereas there have been reports that the endurance of pain is valued as symbolizing a test of character and strength in some African cultures [7]; this did not come out in the sample for the study being reported here. Participants did not seem to attach any value to the pain accompanying male circumcision. Rather, they demanded that an effective pain relief measure should be instituted. Concerns about sexual dis-inhibition and promiscuity associated with male circumcision have also been reported by other researchers [10]. In Malawi, the motivation for circumcising 12-year-old boys has been that boys were able to take care of themselves and to benefit from the counseling that accompanied the traditional initiation activities that had circumcision as a component and that sexual restraint was better if circumcision was done before sexual debut [8].

The potential conflict over the claim for circumcision knowledge for modern health care and the traditional cultural practice that participants in this study reported has also been recognized as medical intervention versus a mark of cultural identity in the literature [11]. The issue has been described as a double standard in that some of the countries advocating for neonatal circumcision in Africa were not practicing it [11]. 

Reports from a few participants point to the importance of some factors that have been labelled cues to the action in this report (a concept from the health belief model). Such factors as a sense of self-determination and receiving information from multiple sources acted as “a push” for a decision to circumcise. 

### 3.3. Implications of the Study Findings for Programing 

#### 3.3.1. Devising Innovative Ways of Reaching Communities

While taking services to the people may probably be one of the best ways, it may take some time to be realized. There are more realistic approaches that we may learn from the referral system that is currently operating in the rural areas of Botswana, with of course modifications as may be appropriate. In areas where there is no reliable public transport, local facilities could screen interested people, provide them with counseling and HIV testing, and submit a list of eligible candidates for booking at the treatment centers. The treatment centers will draw a schedule that targets different localities every day of the week and send out a vehicle to pick up candidates and return them after they have had the surgery. The arrangements can relieve people of the stress of transport, housing, and hunger that participants were concerned about. 

#### 3.3.2. Establishment of a Moving Health Clinic

One way of taking service to the people could be through a moving circumcision clinic whereby an appropriately designed and fully equipped and staffed vehicle moves around camping at strategically located community sites to deliver services, goes back for refilling, and then heads to another locality. It is important to note that for the mobile arrangements to be effective, they must have a well-designed client follow-up system. 

#### 3.3.3. The Need to Search for Effective Postoperative Pain Relief Measure

Men undergoing circumcision need an effective postoperative pain relief because pain has been reported as a deterrent to seeking circumcision. Especially in the wake of palliative care, pain control has become an important topic as it has been noted that health workers leave clients to endure the pain that could otherwise be kept under control [12]. Another pattern that has been observed in this study was that some clients were coming for surgery drunk. Alcohol may be a strategy to cope with pain because ordinarily people coming for elective surgery do not come drunk. Overdosing oneself with alcohol should sensitize the staff in male circumcision treatment centers to the gravity of pain that clients are enduring and find effective pain relief measures. 

Challenges related to the surgery itself, such as pain, bleeding, and infection call for more diligence in the conduct of circumcision. A detailed explanation of the procedural steps should be provided to eligible men before the surgery to avoid misconceptions about what to expect. It is also important that circumcised men are thoroughly counseled to reduce their chances of exaggerating the pain and scaring away others. 

Botswana can learn from other countries that are also implementing male circumcision. In Kenya, innovative approaches have been the creation of a master list of trained staff who can be hired on a temporary basis during high-demand periods, the formation of mobile, rapid response service teams that move freely to sites with high demand, the implementation of client prioritization schemes serving males by age category, and encouraging staff to take timeoff during periods of low demand [13]. Tanzania is reported to be using assembly service model with standardized human and material resource allocation using WHO guidelines for improving efficiency [14]. 

#### 3.3.4. Implication of the Study Findings for Research

*(1)* *Need for Research on Traditional Circumcision*. There is a need to research on traditional circumcision especially to unveil the secrecy that surrounds it. Mutual understanding and appreciation could enhance collaboration between the traditional and the modern systems of circumcision. Such questions as the purpose behind the practice, the approach, the benefits, pain management, wound management, and healing can be explored. Participants called for the collaboration between male circumcision and the traditional initiation schools. However, unless the two systems are open with one another, mistrust will prevail and will make collaboration difficult. 

Research on the efficacy of male circumcision in preventing HIV infection needs to be continued. The male circumcision initiative being reported here is mainly informed by three randomized controlled trials in Kenya, Uganda, and South Africa that indicated that male circumcision can reduce the probability of female-to-male HIV infection by 51% to 60%. However, other reports indicate otherwise. In Lesotho, the high prevalence of male circumcision has not significantly explained HIV status, and this has been thought to be a result of failure to control for traditional circumcision that often does not remove the entire foreskin, among other factors [15]. 

In a meta-analysis of 29 published articles, Van Howe [16] concluded that evidence linking circumcised penis with low risk of female-to-male HIV transmission was misleading as there were a number of methodological flaws in reported studies including failure to control for the presence of genital ulcers and differing sample sizes for circumcised and uncircumcised men in single studies. Van Howe's analysis revealed that the risk of acquiring HIV was even higher for circumcised than uncircumcised men. These conflicting reports should sensitize researchers to continue research and to update communities on new discoveries. Educating communities could reduce misconceptions and help them to make informed choices. 

Finally, findings on the challenges of male circumcision and its communication strategy could provide program implementers with insights into why the demand for male circumcision is slow in picking up and therefore guide them in designing and delivering the program in such a way that barriers to its accessibility are minimized. Communities' values and preferences can be taken into consideration in both curriculum and program design. 

## 4. Conclusions

The findings of the study suggest that, although male circumcision is generally acceptable to communities in Botswana [17], there are challenges that frustrate the initiative. The challenges include sociocultural, knowledge/information, and infrastructural and system factors. The authors present some suggestions for addressing some of the challenges or impediments. There is a need for research on cultural issues of male circumcision unique to Botswana. 

The need for adequate resources, effective monitoring system, involvement of local communities in formulating circumcision policies that are aligned to international standards, factoring in of opportunity costs for the enhancement of service accessibility, and effective communication strategy has been underscored as all these factors are important for any male circumcision to work [11]. Openness to learn from others facing similar challenges that Botswana is confronted with is encouraged. The findings of the study can inform male circumcision program development and implementation in that the challenges can be anticipated for different population sectors and taken into consideration where necessary. 
